# Chronic Kidney Disease Induced by Cisplatin, Folic Acid and Renal Ischemia Reperfusion Induces Anemia and Promotes GATA-2 Activation in Mice

**DOI:** 10.3390/biomedicines9070769

**Published:** 2021-07-02

**Authors:** Gabriel Rufino Estrela, Leandro Ceotto Freitas-Lima, Alexandre Budu, Adriano Cleis de Arruda, Mauro Sergio Perilhão, Ricardo Ambrósio Fock, Jonatan Barrera-Chimal, Ronaldo Carvalho Araújo

**Affiliations:** 1 Department of Clinical and Experimental Oncology, Hematology and Hematotherapy Discipline, Federal University of São Paulo, São Paulo 04037-002, Brazil; 2 Department of Medicine, Nephrology Discipline, Federal University of São Paulo, São Paulo 04039-032, Brazil; adriano.arruda@unifesp.br (A.C.d.A.); maurospersonal3@gmail.com (M.S.P.); 3 Department of Biophysics, Federal University of São Paulo, São Paulo 04039-032, Brazil; lcf.lima@gmail.com (L.C.F.-L.); alexandre.budu@unifesp.br (A.B.); 4 Department of Clinical and Toxicological Analysis, Faculty of Pharmaceutical Sciences, University of Sâo Paulo, São Paulo 05508-000, Brazil; hemato@usp.br; 5 Institute of Biomedical Research, Autonomous University of Mexico, Ciudad Universitaria, Postal mail 70228, Mexico City 04510, Mexico; jbarrera@biomedicas.unam.mx; 6 Investigation Unity UNAM-INC, National Institute of Cardiology Ignacio Chávez, Mexico City 04360, Mexico

**Keywords:** chronic kidney disease, anemia, experimental disease model

## Abstract

Anemia is a common feature of chronic kidney disease (CKD). It is a process related to erythropoietin deficiency, shortened erythrocyte survival, uremic erythropoiesis inhibitors, and disordered iron homeostasis. Animal models of CKD-induced anemia are missing and would be desirable in order to study anemia mechanisms and facilitate the development of novel therapeutic tools. We induced three different models of CKD in mice and evaluated the development of anemia characteristics. Mice were subjected to unilateral ischemia-reperfusion or received repeated low doses of cisplatin or folic acid to induce nephropathy. Renal function, kidney injury and fibrotic markers were measured to confirm CKD. Moreover, serum hemoglobin, ferritin and erythropoietin were analyzed. Renal mRNA levels of *HIF-2α*, erythropoietin, hepcidin, *GATA-2*, and *GATA-2* target genes were also determined. All three CKD models presented increased levels of creatinine, urea, and proteinuria. Renal up-regulation of *NGAL*, *KIM-1*, and *TNF-α* mRNA levels was observed. Moreover, the three CKD models developed fibrosis and presented increased fibrotic markers and α-SMA protein levels. CKD induced decreased hemoglobin and ferritin levels and increased erythropoietin levels in the serum. Renal tissue showed decreased erythropoietin and *HIF-2α* mRNA levels, while an increase in the iron metabolism regulator hepcidin was observed. *GATA-2* transcription factor (erythropoietin repressor) mRNA levels were increased in all CKD models, as well as its target genes. We established three models of CKD-induced anemia, regardless of the mechanism and severity of kidney injury.

## 1. Introduction

The prevalence of chronic kidney disease (CKD) is about 13% worldwide, which accounts for an estimated 850 million affected individuals [1]. It has been recognized that a patient who survives from acute kidney injury (AKI) may develop CKD [2,3,4]. When an acute insult occurs, several tissue repair mechanisms are capable of restoring renal function. This wound healing process consists of consecutive events, such as acute inflammation and resolution, extracellular matrix synthesis (fibrinogenesis), epithelial cell de-differentiation, proliferation, and repair of the tubular structure [5,6,7]. If the repair mechanisms are interrupted, defective or the injury-causing stimulus persists, AKI can progress to a chronic disorder, characterized by an irreversible remodeling of the organ, leading to fibrosis and dysfunction [2,3,6]. Moreover, recent single nucleus sequencing studies identified that after an acute kidney injury, a subpopulation of proximal tubular epithelial cells fail to repair, thus increasing the expression of pro-inflammatory and pro-fibrotic mediators, contributing to fibrogenesis and CKD progression [8]. Histologically, this process presents itself with glomerulosclerosis, vascular rarefaction, and tubulointerstitial fibrosis [4]. The excessive deposition of the extracellular matrix, particularly the presence of collagen fibers, is the most striking feature of tubulointerstitial fibrosis [4,9]. In fibrotic kidneys, interstitial spaces are filled with fibrillar material constituted of fibronectin, type I, and type III collagen [10,11]. 

Anemia is commonly observed in CKD patients and is associated with poor renal and cardiovascular outcomes [12]. Damaged kidneys produce less erythropoietin (EPO), an erythropoiesis stimulating factor, which results in lower production of red blood cells in the bone marrow [12]. EPO deficiency is a determinant cause of anemia in CKD, as the kidney is the main source of EPO production [12]. Iron deficiency anemia is also noticed in CKD patients, and it is typically defined by low serum ferritin levels [13,14]. Moreover, the overproduction of hepcidin, a hormone responsible for maintaining systemic iron homeostasis, which is produced by the liver and secreted into circulation, may explain the impaired iron absorption observed in many CKD patients [15,16,17]. Pro-inflammatory cytokines induce hepcidin, as a mechanism to sequester iron from invading pathogens, leading to iron sequestration, iron deficiency, and anemia [12,17]. 

Better knowledge on the molecular mechanisms underlying anemia in CKD can help in the development of new pharmacologic agents. Currently, animal models of CKD that develop anemia characteristics and allow the exploration of anemia mechanisms in CKD are few [18]. Therefore, the development and characterization of new CKD animal models developing anemia will facilitate the exploration and understanding of the mechanisms of this disease.

## 2. Methods

### 2.1. Animals 

Male C57BL/6 mice weighing 22–27 g and aged 9–12 weeks were used for these experiments. The animals were obtained from the Animal Care Facility of the Federal University of São Paulo (UNIFESP). All animals were housed in individual, standard cages and had free access to water and food. All procedures were previously reviewed and approved by the internal ethical committee of the Federal University of São Paulo in accordance with rules issued by the National Council for Control of Animal Experimentation (CONCEA), the project was approved in 24 August 2019, protocol number CEUA 3456260419.

### 2.2. Experimental Protocol 

The mice were divided into the following groups for each experiment: control group, folic acid-treated group (FA), cisplatin-treated group (CIS), and ischemia reperfusion group (IR). We used *n* = 6–8 for each experiment and condition.

### 2.3. Ischemia Reperfusion

The mice were anesthetized with ketamin (91 mg/kg) and xylazin (9.1 mg/kg) i.p before surgical procedure. A unilateral flank incision was performed to expose the kidney, and the renal pedicle was dissected. The incision was made only in the left flank. We induced renal ischemia by placing non-traumatic vascular clamps over the dissected left renal pedicles for 30 min. The clamps were then released, and the mice received 0.5 mL of 0.9% NaCl (37 °C). Incisions were closed in two layers, with 5–0 sutures, and reperfusion was allowed. The mice were followed for 6 weeks. Twenty-four hours before the euthanasia, mice were subjected to nephrectomy of the right kidney to evidence renal dysfunction. For the nephrectomy, the same procedures listed before were employed, the right kidney was exposed and then two ligatures (5–0 silk) were placed around the renal vessels, each one with a single knot, the kidney was removed, and incisions were closed as previously mentioned. Sham treated mice were subjected to the same procedure, but without renal pedicle clamping. 

### 2.4. Folic Acid Nephropathy 

A single intraperitoneal injection of folic acid (250 mg/kg—Sigma-Aldrich, San Luis, MO, USA) in 0.3 M NaHCO3 vehicle or vehicle alone was administered. Mice were euthanized 28 days after folic acid injection.

### 2.5. Cisplatin Treatment 

Cisplatin treatment consisted of repeated dosing with one dose of cisplatin (7 mg/kg—Bergamo, Taboão da Serra, Brazil) intraperitoneally (i.p.) per week for four weeks. Mice were euthanized 30 days after the last cisplatin injection. 0.9% NaCl was used as vehicle for control mice.

### 2.6. Blood Sampling and Tissue Collection 

The mice were anesthetized with ketamin (91 mg/kg) and xylazin (9.1 mg/kg) i.p. and blood was collected via cardiac puncture. For serum, blood samples were allowed to clot for 2 h at room temperature and then centrifuged for 20 min at 2000× *g*. The samples were then stored at −20 °C. Kidney tissue was collected, and the renal capsule was removed. Transversal cuts were performed, and the kidneys were immediately frozen in nitrogen and then stored at −80 °C. 

### 2.7. Renal Function 

Serum creatinine and urea levels were used to determine renal function. Samples were analyzed using commercially available colorimetric assay kits (Labtest, Lagoa Santa, Brazil). Urine was collected in metabolic cages over 24 h three days prior to euthanasia and protein concentration was determined with Sensiprot assay kit (Labtest, Lagoa Santa, Brazil).

### 2.8. Hemoglobin Analysis 

The HemoCue Hb 301 portable system for hemoglobinometry can measure Hb concentration in less than one minute. Briefly, this device measures Hb by spectrophotometry using a small volume (tailcut) optical measuring cuvette and short light path. This device allows the measurement of Hb concentration in blood samples with low sample volume.

### 2.9. Blood Count Test

Blood samples were collected with EDTA (Merck, Darmstadt, Germany), and blood counts were performed using ABX Micros ABC Vet^®^ equipment (Horiba ABX, Montpellier, France). 

### 2.10. Real-Time PCR 

Kidney samples were frozen at −80 °C immediately after collection. Total RNA was isolated using TRIzol Reagent (Invitrogen, Carlsbad, CA, USA). RNA integrity was assessed by electrophoresis on an agarose gel. cDNA was synthesized using the “High Capacity cDNA Reverse Transcription Kit” (Applied Biosystems, Waltham, MA, USA). Standard curves were plotted to determine the amplification efficiency for each primer pair. Real-time PCR was performed using SYBR Green system (Thermo Scientific, Waltham, MA, USA) using specific primers for *β-actin, 18S, EPO, HIF2a, hepcidin, collagen1A1, α-SMA, TGF-β, fibronectin*, *TNF*-α, *NGAL, KIM-1, GATA2, ADORA3, NRG-1*, and *SELE*; the primers were designed using primer3 web and their specificity was confirmed using NCBI primer-BLAST and then synthesized (Exxtend, Campinas, Brazil); their sequences are listed in Table 1. The cycling conditions were as follows: 10 min at 95 °C, followed by 45 cycles of 30 s at 95 °C, 30 s at 60 °C, and 30 s at 72 °C. Target mRNA expression was normalized to β-actin and 18s and expressed as a relative value using the comparative threshold cycle (Ct) method (2−ΔΔCt). The expression levels of the genes of interest were normalized to the control group and presented as fold change.

### 2.11. Enzyme-Linked Immunosorbent Assay 

Serum samples were frozen and stored at −20 °C immediately after collection. Serum levels of EPO (KA1998) and Ferritin (KA1941) were quantified using ELISA mouse kits specific for each analyte (Novus Biologicals, Littleton, CO, USA), according to the manufacturer’s instructions.

### 2.12. Renal Fibrosis Analysis

The kidneys were fixed in formaldehyde 10% and then dehydrated and embedded in paraffin. Sections (4 µm) were cut and stained with hematoxylin eosin and sirius red. At least six subcortical fields were visualized and analyzed for each mouse using a Leica DM4000 microscope at a magnification of 200×. Tubular injury score was determined based on the percentage of tubules showing luminal casts, cell detachment, or dilation and assigned according to the following scale: 0 = 0 to 5%, 1 = 6 to 25%, 2 = 26 to 50%, 3 = 51 to 75%, and 4 > 75%. Histology analysis was performed blind to experimental groups to assess tubule-interstitial fibrosis based on the sirius red-positive area and assigned according to the following scale: 1 ≤ 25%, 2 = 26 to 50%, 3 = 51 to 75%, and 4 > 75%. 

### 2.13. Kidney Extraction and Sectioning 

The kidney was collected, and then cryoprotected for 2 additional days by immersion in 30% sucrose at 4 °C. The kidney was sectioned at 7 μm with a cryostat (Leica Biosystem, Wetzlar, Germany) at −24 °C. A series of 4 coronal sections of the kidney was mounted for immunofluorescence analysis.

### 2.14. Immunofluorescence 

Immunofluorescence for α-SMA was performed by incubating the sections with Alexa Fluor 488 anti-rabbit (1:300, ThermoFisher, Waltham, MA, USA, #A11034) and anti-mouse sections. The nuclei were stained with DAPI (1:2000, ThermoFisher, Waltham, MA, USA, #D1306). Samples were incubated with primary mouse anti-α-Smooth Muscle Actin antibody (1:200, Cell Signaling, Danvers, MA, USA, #19245S) overnight at 4 °C. Non-specific binding was controlled by replacing a negative control with the primary antibody. The slices were coverslipped in Mowiol mounting media. Sections were imaged in Zeiss fluorescence microscope (Zeiss, Oberkochen, Germany) using a 488 nm excitation. Fluorescence intensity was analyzed using ImageProPlus software v.4.0 (Media Cybernetics, Rockville, MD, USA) and the results were presented as fluorescence intensity/ area. It is worth noting that all these procedures were performed in a double-blind manner.

### 2.15. Statistical Analysis 

All data are presented as mean ± s.e.m. Comparisons between two groups were conducted using the two-tailed t test when the data were normally distributed. The value for statistical significance was established at *p* < 0.05. All statistical analyses were performed using GraphPad Prism 8 v0.2 (GraphPad, La Jolla, CA, USA).

## 3. Results

### 3.1. Folic Acid, Cisplatin and Ischemia-Reperfusion Lead to Renal Dysfunction

**Figure 1:**
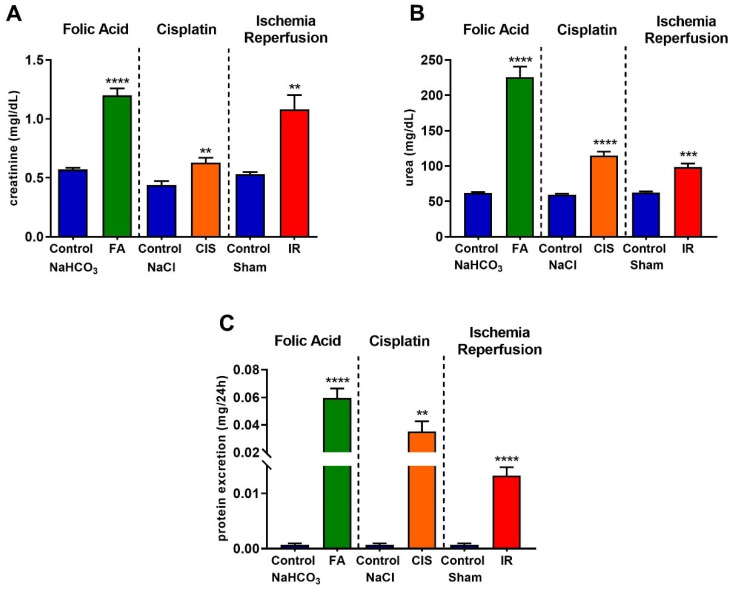
**Renal function in different models of chronic kidney disease.** Renal function was determined by serum creatinine (**A**), urea (**B**), and protein excretion (**C**) after folic acid (FA) treatment, cisplatin (CIS) treatment, and ischemia reperfusion (IR) surgery. Data presented as mean ± SEM; *n* = 6–8. Two-tailed Student´s t-test. ** *p* < 0.01; *** *p* < 0.001; **** *p* < 0.0001.

We performed three different models to induce CKD. The establishment of CKD was confirmed in all three models, as evidenced by the significant increases in plasma creatinine (Figure 1A), urea (Figure 1B), and proteinuria (Figure 1C) levels in the folic acid- and cisplatin-treated mice, as well as in those with CKD induced by ischemia-reperfusion.

### 3.2. Folic Acid, Cisplatin and Ischemia-Reperfusion Increase Kidney Injury Markers

**Figure 2:**
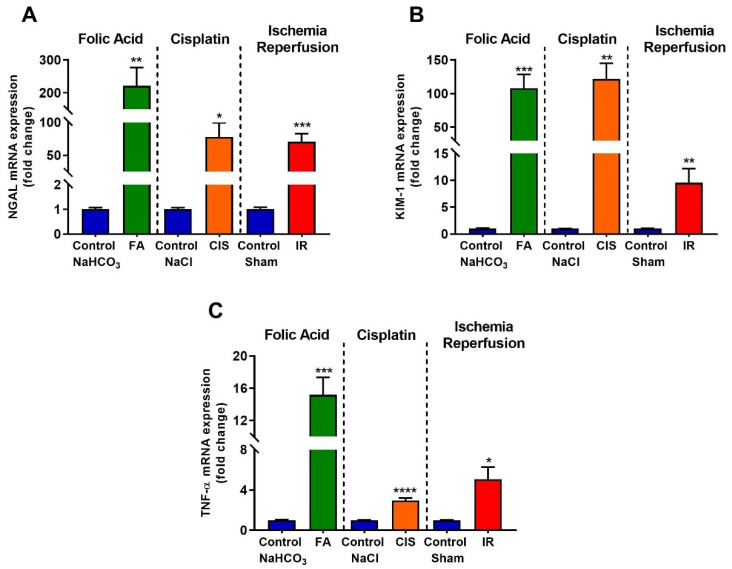
**Chronic kidney disease increases mRNA levels of kidney injury markers.** Renal injury was confirmed by increased mRNA levels of *NGAL* (**A**), *KIM-1* (**B**), and *TNF-α* (**C**) after folic acid (FA) treatment, cisplatin (CIS) treatment, and ischemia reperfusion (IR) surgery. Data presented as mean ± SEM; *n* = 6. Two-tailed Student’s t-test. * *p* < 0.05; ** *p* < 0.01; *** *p* < 0.001; **** *p* < 0.0001.

Real-time PCR was performed to identify tubular injury molecules; the renal expression of neutrophil gelatinase-associated lipocalin (NGAL), which is increased after kidney injury, was up-regulated in our three CKD models (Figure 2A). Moreover, kidney injury molecule-1 (KIM-1), which is a useful biomarker for renal proximal tubule injury, was significantly induced in the renal tissue of folic acid, cisplatin, and ischemia-reperfusion mice, as compared to their respective controls (Figure 2B). Tumor necrosis factor-alpha (TNF-α) is a cytokine produced mainly in macrophages and is increased under pathological conditions and CKD progression. The three mouse models of CKD showed increased mRNA levels of *TNF-α* in the kidney (Figure 2C).

### 3.3. Folic Acid, Cisplatin and Ischemia-Reperfusion Increase Fibrotic Markers

**Figure 3:**
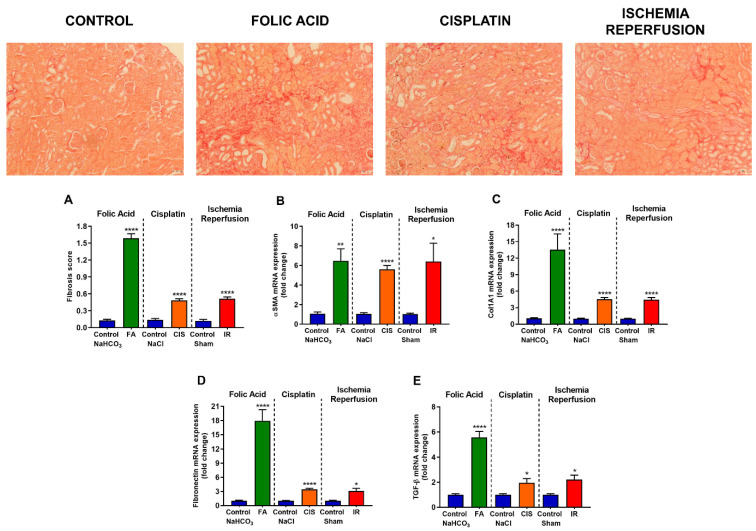
**Fibrosis and mRNA levels of fibrotic markers.** Representative photomicrography of picrosirius red staining at 10x magnification. Fibrosis score (**A**) and mRNA levels of fibrotic markers in renal tissue (**B**–**E**) after folic acid (FA) treatment, cisplatin (CIS) treatment, and ischemia reperfusion (IR) surgery. Scale bars = 50 µM. Data presented as mean ± SEM; *n* = 6–8. Two-tailed Student´s t-test. * *p* < 0.05; ** *p* < 0.01; **** *p* < 0.0001.

**Figure 4:**
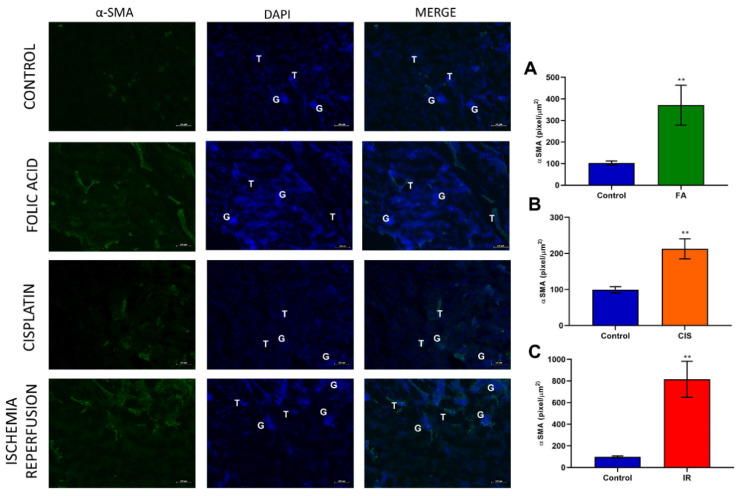
**Effects of fibrogenesis on kidney damage.** The analysis was performed by anti-α-SMA using a fluorescent microscope after different kidney injury protocols. Green color indicates α-SMA positive staining. Blue color indicates nuclei stained with DAPI and the third column indicates the merge of α-SMA and DAPI. A-SMA quantification (**A**–**C**) after folic acid (FA) treatment, cisplatin (CIS) treatment, and ischemia reperfusion (IR) surgery. G indicates glomeruli and a T indicates tubules. Scale bars = 100 µM. Data presented as mean ± SEM; *n* = 6. Two-tailed Student´s t-test. ** *p* < 0.01.

Chronic kidney disease models induced tubulointerstitial fibrosis, as evidenced by the fibrosis score (Figure 3A) obtained from the analysis of the picrosirius red staining slides (Figure 3). Moreover, we analyzed mRNA expression of fibrotic markers such as *α-SMA, Col1A1, Fibronectin*, and *TGF-β*, and all of these genes were upregulated in all three CKD models (Figure 3B–E). We performed immunofluorescence staining for α-SMA in all three CKD models and found an increase of this protein in renal tissue, thus confirming the fibrotic phenotype in our CKD mouse models (Figure 4).

### 3.4. Folic Acid, Cisplatin and Ischemia-Reperfusion CKD Models Develop Anemia

**Figure 5:**
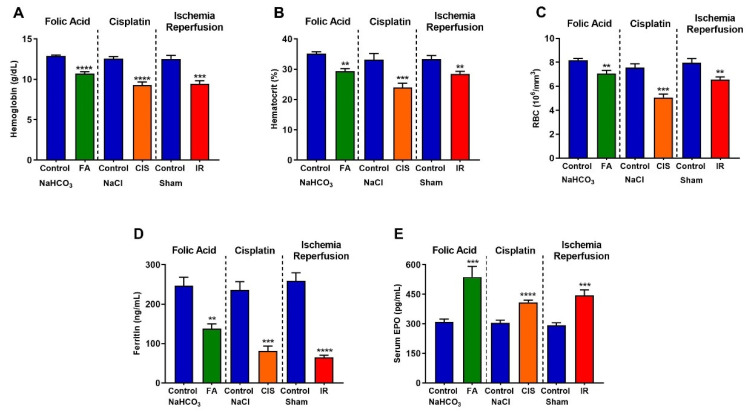
**Chronic kidney disease leads to anemia.** Hemoglobin (**A**), hematocrit (**B**), red blood cells (**C**), serum ferritin (**D**), and serum EPO (E) levels after folic acid (FA) treatment, cisplatin (CIS) treatment, and ischemia reperfusion (IR) surgery. Data presented as mean ± SEM; *n* = 6–8. Two-tailed Student´s t-test. ** *p* < 0.01; *** *p* < 0.001; **** *p* < 0.0001.

Anemia is a common feature of CKD. When assessing hemoglobin levels in our three CKD models, we found a significant reduction in hemoglobin levels (Figure 5A), decreased percentage of hematocrit (Figure 5B), and reduced red blood cell count (Figure 5C) induced by CKD. Moreover, we analyzed serum ferritin levels, which also showed a marked reduction in CKD induced by folic acid, cisplatin, or IR (Figure 5D). Serum erythropoietin levels were analyzed and found to be increased in all three models (Figure 5E). 

### 3.5. Folic Acid, Cisplatin and Ischemia-Reperfusion Decrease Renal Expression of Erythropoietin and Hypoxia Inducible Factor-2 Alpha, and Increase Hepcidin Levels

**Figure 6:**
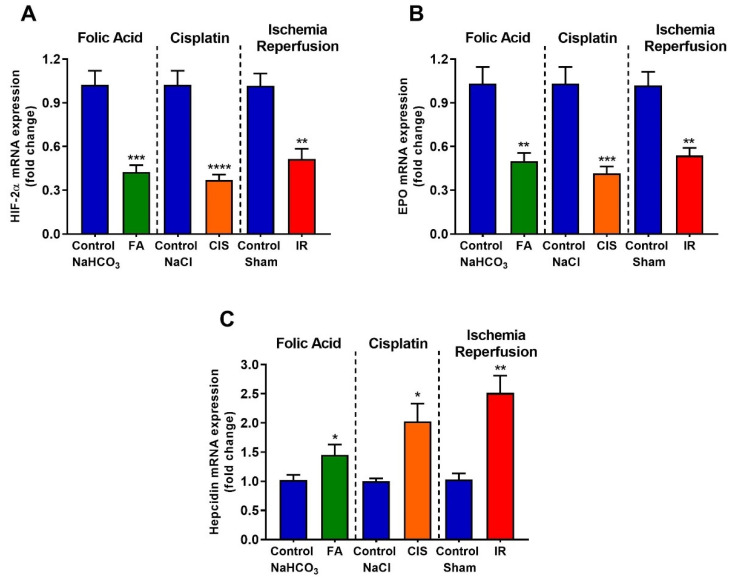
**Effects of CKD on mRNA expression of genes related to red blood cell production and iron regulation.** Renal mRNA expression of *HIF-2α* (**A**), *EPO* (**B**), and *Hepcidin* (**C**) after folic acid (FA) treatment, cisplatin (CIS) treatment, and ischemia reperfusion (IR) surgery. Data presented as mean ± SEM; n = 6. Two-tailed Student´s *t*-test. * *p* < 0.05; ** *p* < 0.01; *** *p* < 0.001; **** *p* < 0.0001.

We performed real-time PCR in renal tissues for anemia-linked genes. Hypoxia-inducible factor (HIF) is a transcription factor that responds under low oxygen availability; specifically, HIF-2α is the main erythropoietin production regulator. All three CKD models presented reduced levels of *HIF-2α* mRNA (Figure 6A). Additionally, renal *EPO* mRNA levels were also reduced in the three CKD mouse models. (Figure 6B). Hepcidin is a hormone that in excess impairs iron absorption and contributes to anemia development. Therefore, we performed qPCR for *hepcidin* in the renal tissue and observed a significant increase in *hepcidin* mRNA levels in the three CKD models (Figure 6C). 

### 3.6. Folic Acid, Cisplatin and Ischemia-Reperfusion Increase GATA-2 Transcription Factor and the Expression of Its Target Genes

**Figure 7:**
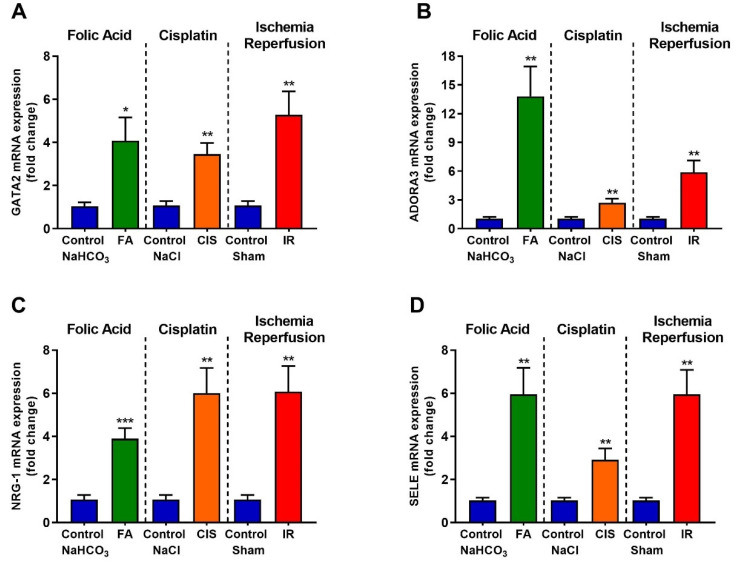
**CKD leads to increased activation of GATA-2.** Renal mRNA expression of *GATA-2* (**A**) and *GATA-2* target genes (**B**–**D**) after folic acid (FA) treatment, cisplatin (CIS) treatment, and ischemia reperfusion (IR) surgery. Data presented as mean ± SEM; *n* = 6. Two-tailed Student’s *t*-test. * *p* < 0.05; ** *p* < 0.01; *** *p* < 0.001.

GATA-2 is a transcription factor which, when activated in renal tubular cells, suppresses *EPO* expression [19,20,21]. Therefore, we tried to analyze the mRNA levels of *GATA-2* in the renal tissue and the mRNA levels of some of its target genes to confirm their activation. We observed increased mRNA levels of *GATA-2* in the kidneys from the three different CKD models (Figure 7A). Moreover, we observed increased mRNA levels of *ADORA3*, *NRG-1*, and *SELE* (*GATA-2* target genes) for all the CKD models analyzed, thus confirming *GATA-2* activation in the kidney of CKD mice (Figure 7B–D).

## 4. Discussion

Anemia has been associated with CKD for about 170 years. While kidney disease progresses, anemia prevalence increases. Anemia in CKD reduces the quality of life and increases the susceptibility to hospitalization and mortality [12]. Understanding the molecular mechanism of anemia in CKD is important for the development of therapeutic tools. Therefore, animal models are of great value to explore and discover targets and new pharmacological approaches.

Here, we used three different CKD models and assessed whether mice developed anemia features. First, we confirmed that our models led to CKD by measuring renal function, for which increased creatinine, urea and proteinuria are standard clinical tests to indicate the development of CKD. Moreover, we showed increased kidney injury markers, such as *NGAL, KIM-1 and TNF-α*. Considering that CKD leads to increased tubulointerstitial fibrosis [4], we analyzed fibrotic markers in the renal tissue, where our models showed increased levels of both mRNA and protein. 

Low hemoglobin levels are a good indicator of anemia; in all our CKD models, we found reduced hemoglobin levels accompanied by low serum ferritin levels, which may indicate iron deficiency. Indeed, hepcidin, a hormone produced in the liver which is responsible for maintaining iron homeostasis [15,16,17], is up-regulated in all our three models. Excess hepcidin is known to impair dietary iron absorption in many CKD patients, thus providing a possible mechanism for anemia induction in our CKD models, moreover NGAL which is commonly used to determine kidney injury, is a mediator of iron sequestration, some studies showed that NGAL participates in the iron-depletion of the innate immune system [22,23]. 

Serum EPO was found to be slightly increased in our three models. Although EPO deficiency is a common predictor of anemia, some studies show that liver and macrophage synthesis of EPO is observed [24,25], moreover this could be an attempt to compensate for reduced EPO synthesis in the kidney [26]. HIF-2α is crucial for the induction of erythropoietin; ablation of HIF-2α in mice leads to anemia due to inadequate production of renal EPO, in addition, the location of renal EPO-producing cells coinciding with the location of renal interstitial HIF-2α-expressing cells [27,28]. Indeed, we observed reduced *HIF-2α* and *EPO* renal expression in all our three models, corroborating these findings. *GATA-2* or *GATA-binding factor 2* is a transcription factor that regulates genes expression. Reduced GATA-2 results in the stimulation of EPO gene transcription. On the other hand, increased TNF-α induces GATA-2 activity and this, in turn, reduces EPO gene transcription [19,20,21,29]. Here, we observed increased *TNF-α* levels, associated with increased *GATA-2* expression and activation, as evidenced by the increase in its target genes, which may also explain the low renal expression of *EPO*. 

In summary, we showed that our three CKD models are capable of inducing anemia, regardless of the mechanisms and severity of the kidney injury. Moreover, we showed that renal *GATA-2* and its target genes are increased in CKD, which might explain the reduced renal expression of *HIF-2α* and *EPO*. In addition, since *TNF-α* levels are increased by CKD, this might be the link between CKD and increased GATA-2 activity. Another mechanism that might contribute to anemia development and that was common for the three CKD models was the increased renal hepcidin levels. Here, we established three new models for the study of CKD-induced anemia in mice, opening the possibility to further explore strategies to avoid or reverse these effects, and providing one reliable tool to explore the molecular mechanism behind this disease. 

## Figures and Tables

**Table 1:** Sequences of the primers used for real-time PCR assays.

Primers for RT-PCR		
Gene	Forward 5’-3’	Reverse 5’-3’
18S	CGC CGC TAG AGG TGA AAT TC	TCT TGG CAA ATG CTT TCG C
β-actin	CTG GCC TCA CTG TCC ACC TT	CGG ACT CAT CGT ACT CCT GCT T
NGAL	ATG TGC AAG TGG CCA CCA CG	CGC ATC CCA GTC AGC CAC AC
TNF-α	GCC TCT TCT CAT TCC TGC TTG	CTG ATG AGA GGG AGG CCA TT
KIM-1	TGT CGA GTG GAG ATT CCT GGA TGG T	GGT CTT CCT GTA GCT GTG GGC C
TGF-β1	CAA CAA TTC CTG GCG TTA CCT TGG	GAA AGC CCT GTA TTC CGT CTC CTT
Col1A1	CCC CGG GAC TCC TGG ACT T	GCT CCG ACA CGC CCT CTC TC
α-SMA	TTG GAA AAG ATC TGG CAC CAC	GCA GTA GTC ACG AAG GAA TAG
Fibronectin	CCT ACG GCC ACT GTG TCA CC	AGT CTG GGT CAC GGC TGT CT
HIF-2α	CTG GAC AAA GCC TCC ATC AT	TTG CTG ATG TTT TCC GAC AG
EPO	TCC ACT CCG AAC ACT CAC A	CCT CTC CCG TGT ACA GCT T
Hepcidin	TCC TGC TTC TCC TCC TTG C	GCT TTC TTC CCC GTG CAA A
GATA2	ATC TCT TCG GCT TCC CAC C	TTG ACG CCA TCC TTG TCC T
ADORA3	GAA CCC CAC TCT GAG GAC C	GCA ATG GCC AAA GGT GTG A
NRG-1	CTA CCC ACC TTG ACC CTG G	TGA GAC CAG AAC AGC GGA G
SELE	GAG TTT CAC GTT GCA GGG G	GGC GCA GAT AAG GCT TCA C

## Data Availability

All data generated for this study is in this article.

## Abbreviations

αSMA	Alpha smooth muscle actin
ADORA3	Adenosine A3 receptor
CKD	Chronic Kidney Disease
CIS	Cisplatin
COL1A1	Collagen 1A1
EPO	Erythropoietin
FA	Folic acid
GATA2	GATA binding factor 2 or erythroid transcription factor
HIF2α	Hypoxia-inducible factor 2
IR	Ischemia reperfusion
KIM-1	Kidney injury molecule-1
NGAL	Neutrophil gelatinase-associated lipocalin
NRG-1	neuregulin 1
SELE	Selectin E
TGF-β	Transforming growth factor beta
TNF-α	Tumor necrosis factor alpha
